# Early vs. late MRD response- and risk-based treatment intensification of childhood acute lymphoblastic leukemia: a prospective pilot study from Saudi Arabia

**DOI:** 10.1186/s40164-018-0121-x

**Published:** 2018-11-19

**Authors:** Wasil Jastaniah, Naglla Elimam, Khalid Abdalla, Aeshah A. AlAzmi, Aml M. Elgaml, Ahmad Alkassar, Mustafa Daghistani, Sami Felimban

**Affiliations:** 1 Department of Pediatrics, Faculty of Medicine, Umm AlQura University, Makkah, Saudi Arabia; 2 Princess Noorah Oncology Center, King Saud Bin Abdulaziz University for Health Sciences and King Abdulaziz Medical City, Ministry of National Guard Health Affairs, P.O. Box 9515, Jeddah, 21423 Saudi Arabia; 3 Department of Pharmaceutical Care, Clinical Pharmacy, Pediatric Hematology/Oncology, King Saud Bin Abdulaziz University for Health Sciences and King Abdulaziz Medical City, Ministry of National Guard Health Affairs, Jeddah, Saudi Arabia; 4 Department of Pathology and Laboratory Medicine, Flow Cytometry Unit, King Abdulaziz Medical City, Ministry of National Guard Health Affairs, Jeddah, Saudi Arabia; 5 Department of Pathology and Laboratory Medicine, Cytogenetics and Molecular Cytogenetics Unit, King Abdulaziz Medical City, Ministry of National Guard Health Affairs, Jeddah, Saudi Arabia; 6 College of Applied Medical Sciences, King Saud Bin Abdulaziz University for Health Sciences and King Abdulaziz Medical City, Ministry of National Guard Health Affairs, Jeddah, Saudi Arabia

**Keywords:** Acute lymphoblastic leukemia, Treatment, Stratification, MRD, Response, Risk, Children

## Abstract

**Background:**

Refinement of risk-based treatment stratification by minimal residual disease (MRD) at different time points has improved outcomes of childhood acute lymphoblastic leukemia (ALL). In this prospective study we evaluated effects of such stratification, including intensification of therapy based on response assessment at day-15 and MRD at day-29 of induction to test if treatment intensification would improve outcomes.

**Methods:**

241 patients, 1–14 years old, newly diagnosed with ALL, were recruited and stratified by risk and MRD response into three treatment Arms (A, B, or C). Arm A was modified from COG AALL0331, B from AALL0232, and C from AALL0232 and AALL0434. Assignments were according to NCI risk, phenotype, rapid vs. slow early response (SER), steroid pretreatment, MLL rearrangement (*MLLR*), CNS3, and testicular involvement. Patients on Arm A had treatment intensified early based on day-15 marrow results or late based on end-of-induction MRD.

**Results:**

5-year OS, EFS, and CIR were 89.5% ± 4.0%, 87.6% ± 4.3%, and 7.1% ± 3.5%. No significant difference was found by B- vs. T cell phenotype. 5-year OS, EFS, and CIR for B-cell ALL were 90.5% ± 2.4%, 88.7% ± 2.6%, and 6.4% ± 2.0%. Outcomes for patients with *t*(1;19)/*TCF3*-*PBX1* and *MLLR* were significantly (p ≤ 0.05) worse than for other patients. MRD level at end-of-induction associated with outcomes, but association with a specific MRD value at end-of-induction varied significantly by NCI-risk group. Late treatment intensification based on end-of-induction MRD significantly improved survival outcomes for NCI-SR patients, however, patients with NCI-HR and positive MRD at end-of-induction had significantly inferior outcomes despite intensification. MRD transitions between day-15 and day-29 of induction associated with differences for OS and EFS.

**Conclusions:**

Arm switching to a more intensive protocol had mixed results. Assigning patients by end-of-induction MRD-risk alone did not reflect response kinetics of the different NCI-risk groups. Although late treatment intensification improved outcomes of NCI-SR patients with positive MRD at end-of-induction, further refinement is needed to improve outcomes of NCI-HR with SER. Integration of NCI-risk group with specific MRD value and time point allows more refined treatment stratification.

*Trial Registration* Protocols were approved by King Abdullah International Medical Research Center and Ethics Review Committee RC08053J

## Background

Acute lymphoblastic leukemia (ALL) is the most common childhood malignancy, representing 25% of all childhood cancers. With intensive multi-agent chemotherapy, cure rates for children with ALL now approach 90% [1]. However, racial disparities exist in outcomes, specifically that, in the USA, Hispanic children and young adults are more likely to suffer a negative outcome with ALL [2]. Furthermore, significant differences in long-term event free survival (EFS) exist among patient subgroups. Trials have led to the development of a risk- and response-based classification system that relies on the National Cancer Institute (NCI)—Rome criteria and on presence/absence of central nervous system (CNS), testicular disease, and other biological features that include immunophenotype, cytogenetics, rapidity of response, and minimal residual disease (MRD) as determined by flow cytometry [3]. Despite these advances, a considerable number of children with ALL still relapse.

MRD measurement at different time points and at end-of-induction has been shown to be a powerful informative prognostic predictor of outcome in ALL [4]. Current contemporary protocols incorporate MRD monitoring to stratify treatment intensity [5–8], however, recent evidence suggests that MRD alone is not sufficient to fully predict outcomes [9, 10]. Thus, integration of different prognostic factors with MRD assessment would help ensure optimal treatment stratification, which is a key component towards precision medicine.

The outcome of ALL treatment in Saudi Arabian children has not been prospectively studied. Our aim in this prospective study was to refine treatment and risk stratification based on clinical and genetic features at diagnosis and rapidity and degree of response to induction therapy and to investigate childhood ALL response and toxicity patterns in our population based on risk and MRD-based response stratification. We intensified therapy early (during induction) or late (post-induction) based on risk and response in order to determine if early vs. late MRD-based response and risk-based treatment intensification would improve outcomes.

We recruited a substantial number of children, 1–14 years of age, with ALL under the prospective childhood acute lymphoblastic leukemia 2008 (CALL08) study protocol at the Princess Noorah Oncology Center, King Abdulaziz Medical City, Jeddah, Saudi Arabia. The CALL08 protocol assigned patients to one of three increasing intensity treatment arms, based on clinical and genetic risk factors we describe herein. In addition, patients in the less rigorous two treatment arms were reassigned to the most intense treatment arm based on early and late response assessment during induction. Several prognostic factors emerged, many of which mostly overlapped with treatment (re)assignment criteria. One important finding was that outcomes associated with a specific MRD value at end-of-induction varied significantly by NCI risk group. Furthermore, patients in our study group deviated from certain established outcomes vs. cytogenetic abnormality. We may also have found an association between treatment toxicity and gender.

## Methods

### Patient recruitment

Pediatric (age 1–14 years) patients newly diagnosed between January 2008 and December 2014 with ALL and having had no prior therapy with the exception of steroids were recruited and treated at the Princess Noorah Oncology Center (PNOC), King Abdulaziz Medical City (KAMC), Jeddah, Saudi Arabia. Written informed parental/legal guardian consent was obtained for all patients and is on file. All protocols were approved by the King Abdullah International Medical Research Center and Ethics Review Committee (KAIMRC ref.#:RC08-053/J). Any patient classified as “very high risk”, defined as having any of *BCR*-*ABL1*-fusion transcript (determined by FISH or RT-PCR); *t*(9;22)(q34;q11) cytogenetics; less than 44 chromosomes and/or DNA index < 0.81; induction failure defined as M3 bone marrow on day 29 of induction or ≥ 0.01% MRD at end of consolidation; or *MLL* rearrangement (*MLLR*) with slow early response (SER) after induction, was excluded. SER was defined as 5% blasts or more in the bone marrow on induction day 15 or MRD ≥ 0.01% bone marrow blasts at day 29 (end-of-induction). Rapid Early Response (RER) was defined as M1 (< 5%) bone marrow blasts at day 15 and MRD < 0.01% at end-of-induction.

### Treatment assignment

**Fig. 1:**
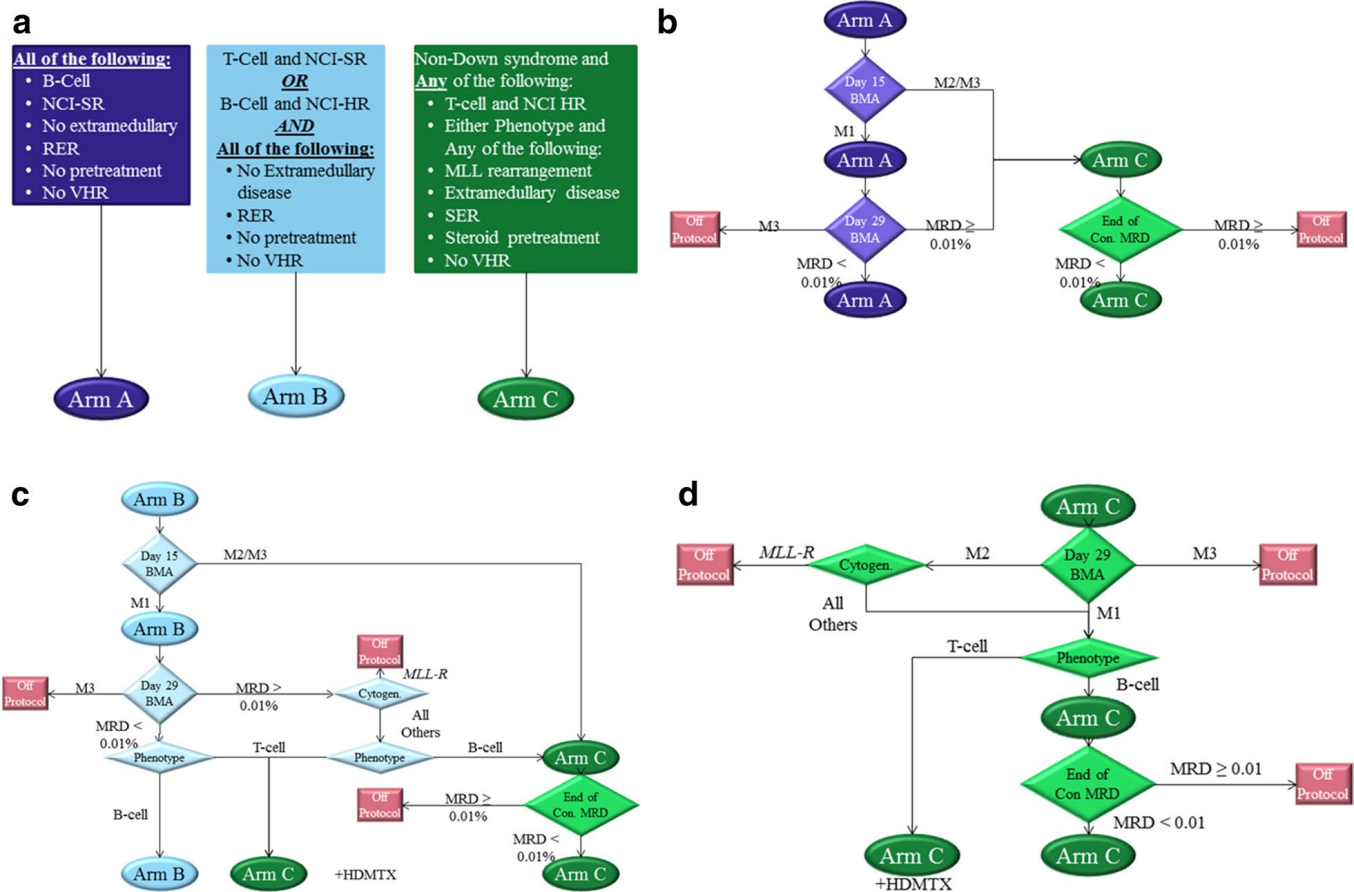
Patient assignment to different protocols. **a** Post-induction treatment assignment criteria (Arm A, Arm B, Arm C). **b** Response-based treatment reassignment algorithm for NCI standard-risk patients initially assigned to induction Arm A. Patients who were M2/M3 (≥ 5% blasts) at day 15 or MRD% ≥ 0.01 at day 29 were reassigned to Arm C. Patients thus reassigned who were still at MRD% ≥ 0.01 at end-of-consolidation were taken off protocol as induction failure. Remaining patients continued on Arm A. **c** Response-based treatment reassignment algorithm for NCI high-risk patients initially assigned to induction Arm B. Induction Arm B patients who were day 29 MRD% ≥ 0.01 were either taken off protocol (if *MLLR*) or reassigned to post-induction Arm C (if B-cell) or Arm C + HDMTX (if T-cell). At end-of-consolidation, patients on Arm C with MRD% ≥ 0.01 were taken off protocol as induction failure. **d** Algorithm for patients initially assigned to induction Arm C or switched from induction Arm A or B to Arm C. Arm C patients who were M3 on day 29 were taken off protocol as induction failure. M2 patients who also had MLLR were also taken off protocol. T-cell patients were supplemented with HDMTX. The remainder continued on Arm C until end-of-consolidation, whereupon those who were MRD% ≥ 0.01 were taken off protocol

Patients were assigned to one of three treatment arms based on risk and response assessment (Fig. 1a). Arm A was assigned to patients who had all of (1) NCI standard-risk (NCI-SR; age 1 to < 10 years, WBC < 50); (2) no extra-medullary (CNS3 or testicular) disease; (3) no steroid pretreatment; (4) No *MLLR*; (5) B-cell immunophenotype; and (6) rapid early response. Patients were later reassessed for final treatment (re)assignment. At day-15, any patient with M2/M3 marrow was reassigned to Arm C. If remaining Arm A patients had day-29 MRD ≥ 0.01%, they were reassigned to Arm C consolidation and re-evaluated at end-of-consolidation. Those who still had MRD ≥ 0.01% post consolidation were taken off protocol. Those who attained MRD ≤ 0.01% were continued on Arm C (Fig. 1b).

Arm B patients had either (1) NCI high-risk (NCI-HR; age ≥ 10 years and/or WBC ≥ 50), B-cell immunophenotype, and RER; or (2) T-cell immunophenotype, NCI-SR, and RER. Patients with marrow M2/M3 status at day-15 were reassigned to Arm C. Patients who continued on Arm B were reassessed at day-29. Any still with MRD ≥ 0.01% and *MLLR* were taken off protocol. Remaining MRD29 ≥ 0.01% patients were reassigned to Arm C; those with T-cell ALL were also dosed with high-dose methotrexate (HDMTX). Of these patients, any who failed to attain MRD < 0.01% by end-of-consolidation were taken off protocol. Patients with T cell ALL and a MRD < 0.01% who were NCI-HR were reassigned to Arm C + HDMTX (Fig. 1c).

Patients were assigned to Arm C/C + HDMTX if they had any of (1) NCI-HR and T-cell immunophenotype (+ HDMTX); (2) T-cell immunophenotype and SER (+ HDMTX); (3) B-cell immunophenotype with SER; (4) *MLLR* with RER; (5) Testicular disease; (6) CNS3 status; or (7) Steroid pretreatment. However, Arm C assignment was not performed for patients with Down syndrome. At day-29, any patients who remained at M3 were taken off protocol. Any with evidence of SER (M2 at day-15 or MRD day-29 ≥ 0.01%) and were *MLLR* ALL were taken off protocol. All remaining T-cell patients on Arm C were supplemented with HDMTX. Those B-cell patients who were still MRD ≥ 0.01% after consolidation were taken off protocol (Fig. 1d).

### Treatment protocols

Arm A consisted of standard 3-drug induction with dexamethasone, PEG asparaginase, and vincristine with 3 intrathecal treatments at day 1, 15, and 29 for CNS1, and for patients with CNS2 an additional two intrathecal doses were given on day 8 and 22. Bone marrow assessment was done on day-15 and at the end-of-induction. End-of-induction bone marrow was subject to MRD analysis and protocol (re)assignment. This protocol used dexamethasone as the steroid in all phases of therapy and intrathecal methotrexate (ITMTX) alone as the standard intrathecal therapy. Patients who remained on this arm continued therapy based on modifications from the COG AALL0331 protocol with standard escalating intravenous Capizzi methotrexate in interim-maintenance phase [11, 12].

Arm B began with a 4-drug induction that included dexamethasone, vincristine, PEG asparaginase, and daunorubicin with 3 intrathecal treatments at day 1, 15, and 29 for CNS1 and for patients with CNS2 two intrathecal methotrexate doses on day 8 and 22 were added. Bone marrow assessment was done on day-15 and at the end-of-induction. End-of-induction bone marrow was also subjected to MRD analysis for final risk classification and protocol (re)assignment. Arm B used dexamethasone as the steroid in all phases and ITMTX as the standard intrathecal therapy with standard escalating intravenous Capizzi methotrexate in interim-maintenance phase based on modifications from the COG AALL0232 protocol [8, 11].

Arm C used an extended augmented BFM-backbone to treat these high-risk patients. HDMTX instead of escalating dose (Capizzi) methotrexate during interim-maintenance-1 was used for T-cell patients with NCI-HR criteria at diagnosis or T-cell patients with SER regardless of NCI-risk based on modifications from the COG AALL0232 and COG AALL0434 protocol [8, 13].

### Down syndrome (DS) patients

DS patients were treated with Arm A or Arm B for NCI-SR or NCI-HR, respectively. Capizzi methotrexate was used during interim-maintenance. Irradiation therapy was used for testicular disease and CNS3 status. Additional modifications included leucovorin rescue after every dose of ITMTX during all phases of therapy except maintenance. SER DS patients continued on Arm B with a single interim-maintenance and delayed-intensification unless considered induction failure. Induction failure DS patients were taken off protocol.

In summary, Arm B used single delayed intensification and single interim maintenance post-induction therapy and Arm C used double delayed intensification and double interim maintenance post-induction therapy with/without high-dose methotrexate in the first interim maintenance phase as detailed above.

### Minimal residual disease and cytogenetic studies

Bone marrow aspirate samples were obtained at diagnosis, at day 15 of induction, at the end-of-induction (day 29), and at the end-of-consolidation if day 29 showed evidence of residual leukemia. Diagnosis of ALL was based on standard morphologic, immunophenotype and genetic studies. Immunophenotyping by flow cytometry was performed on all samples at diagnosis using a standard panel of antibodies. Antibodies were obtained from Becton–Dickinson (San Jose, CA, USA) and Beckman Coulter (Beckman-Coulter, Miami, FL). The flow cytometers used were FC500 cytometers (Beckman-Coulter) and BD FACSCanto II (BD Biosciences, San Diego, CA, USA). MRD assessments from sample preparation to data analysis were performed using standardized validated operating procedures adopted from published studies [14–16].

From January 2008 to May 2009, MRD studies were performed using 6-color panel on FC500 cytometers (Beckman Coulter), 6-color panel from June 2009 to November 2012 and 8-color panel until the end of the study in December 2014 using BD FACSCanto II cytometers. Leukemia-associated immunophenotypes (LAIP) were studied at diagnosis using panels that include the following monoclonal fluorochrome-conjugated antibody combinations: CD58, CD10, CD19, CD34, CD20, CD15, CD13 + CD33, CD81, and CD45 for B-cell precursor ALL; and TdT, CD2, sCD3, cyCD3, CD4, CD5, CD7, CD8, C34, CD45, and CD99 for T-ALL. The same antibody combinations were applied during follow-up for MRD detection for each patient. Data acquisition and analysis was performed on FC500 and BD FACSCanto II flow cytometers and software. At least 30,000 events were acquired and analyzed for identification of LAIP at diagnosis, and at least 300,000 events were required for MRD measurements. The strategy for MRD detection was based on detection of at least 20 clustered events displaying LAIP characteristics. A detection limit of 0.01% (10/100,000 cells) was the threshold limit in all samples and results below the 0.01% threshold were reported as no evidence of residual leukemia or MRD negative.

Standard conventional karyotyping on bone marrow samples at diagnosis was performed, and the International System of Human Cytogenetic Nomenclature was used to describe karyotypes [17]. We also tested *BCR*-*ABL1* fusion transcript by fluorescence in situ hybridization (FISH) or by RT-PCR. FISH for double trisomy involving chromosomes 4 and 10, *MLLR*, *ETV6*-*RUNX1* (or *TEL*-*AML1*) fusion, intrachromosomal amplification of chromosome 21 (iAMP21), and *TCF3*-*PBX1* fusion was tested in patients with B-cell ALL.

### Statistical analysis

Outcome data were tested by Cox proportional hazards models when possible (at least 1 “event” attached to each factor level) or by Kaplan–Meier likelihood ratio test when a factor level did not have any events (e.g., if 5-year CIR = 0 for a specific treatment protocol). Hazard ratios were as calculated by Cox models or, in the case of zero events, calculated with an addition of 0.5 pseudocounts to each cell of the matrix. The p values were calculated by omnibus Anova (likelihood ratio) followed by post hoc multiple range tests. For treatment with covariate tests, not all pairwise comparisons were tested. Instead, contrasts were set up to test all treatments within a specific covariate level and all covariate effects for a specific treatment level. If one of these did not overlap, any pairwise comparison would lack meaning. Two p value cutoffs are reported, p ≤ 0.05 and p ≤ 0.10. While conventional wisdom holds that pairwise tests are not to be done if an omnibus test does not meet at least p ≤ 0.05, this is only necessary if the pairwise test is akin to the least-significant difference (LSD) [18].

## Results

### Demographics

A total of 241 children, aged 1–14 years were diagnosed with ALL and eligible for study entry. Of these, 4 (1.7%) with the Philadelphia cytogenetic abnormality were excluded. The clinical demographics are summarized in Table 1. B-cell ALL accounted for 82.7% of patients and T-cell ALL 17.3%. Induction and post-induction treatment assignment for B-cell ALL is shown in Table 2.

**Table 1:** Patient characteristics

Variable	Total	%	Mean/median	Range
Age (years)			5.77/5.16	1.23–13.35
< 10	211	87.6		
≥ 10	30	12.4		
WBC			76.5/17	0.4–880
< 50 × 10^9^	161	66.8		
≥ 50 × 10^9^	80	33.2		
Gender				
Female	103	43.5		
Male	134	56.5		
Down syndrome				
No	224	94.5		
Yes	13	5.5		
NCI risk				
Standard	145	61.2		
High	92	38.8		
Steroid pretreatment				
No	231	97.5		
Yes	6	2.5		
Immunophenotype				
B-cell	196	82.7		
T-cell	41	17.3		
CNS status				
CNS1	185	78.1		
CNS2	26	11.0		
CNS3	24	10.1		
Missing	2	0.8		
Testes (male only)				
Not involved	131	97.8		
Involved	3	2.2		
Cytogenetic group				
Normal	41	17.3		
Hyperdiploidy	64	27.0		
Double trisomy (+)	36^a^			
Double trisomy (−)	28^a^			
ETV6/RUNX1	23	9.7		
iAMP(21)	5^b^			
*t*(1;19)	6	2.5		
*MLLR*	9	3.8		
Other	81	34.2		
Hypodiploidy (< 44)	0	0.0		
Not available	9	3.8		
Philadelphia	4	1.7		

^a^HD double trisomy (+) and (−) were combined into a single hyperdiploidy (HD) group for analysis

^b^iAMP(21) was combined with “Others” group for analysis

**Table 2:** Treatment assignment and response assessment, B cell ALL (n = 196)

Variable	Value	Count	%
Induction arm^a^	A	125	63.8
	B	50	25.5
	C	21	10.7
Day 15 BM morphology	M1	182	92.9
	M2	8	4.1
	M3	5	2.6
	Missing	1	0.5
Day 15 BM MRD (%)	< 0.01	84	42.9
	≥ 0.01	50	25.5
	Not available	62	31.6
Day 29 BM morphology	M1	191	97.4
	M2	1	0.5
	M3	2	1.0
	Not available	2	1.0
Day 29 BM MRD (%)	< 0.01 (negative)	177	90.3
	≥ 0.01 (positive)	13	6.6
	Not determined	6	3.1
Post induction arm^b^	A	114	58.2
	B	38	19.4
	C	40	20.4
	Not applicable	4	2.0

^a^Patients with Philadelphia translocation positive were excluded from the analysis (n = 4)

^b^4 patients were not eligible for post-induction analysis and were reported as “Not applicable”. Post-induction Arms C and C + HDMTX were combined

### Study outcomes

**Fig. 2:**
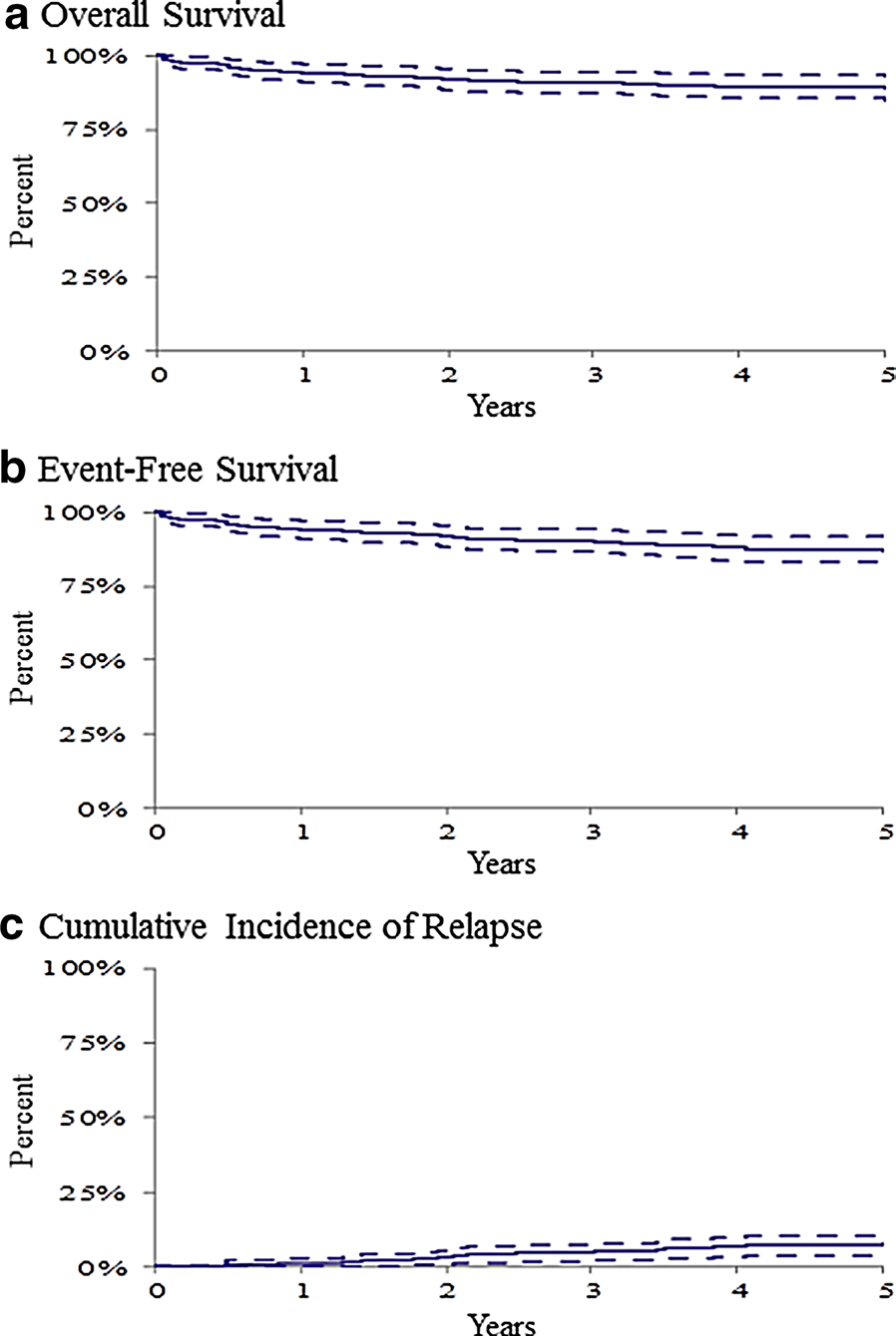
The 5-year overall survival (**a**), event-free survival (**b**), and cumulative incidence of relapse for the entire patient group

The overall 5-year OS, EFS, and CIR were 89.5% ± 4.0%, 87.6% ± 4.3%, and 7.1% ± 3.5% (Table 3, Fig. 2). 5-year OS, EFS, and CIR for B-cell ALL were 90.5% ± 2.4%, 88.7% ± 2.6%, and 6.4% ± 2.0%. 5-year OS, EFS, and CIR for T-cell ALL were 86.0% ± 6.2%, 83.2% ± 7.2%, and 7.4% ± 4.0% (Table 3). No significant difference in survival was found by B vs. T-cell phenotype. As the number of patients with T-cell ALL was small, further analyses were, for the most part, presented for B-cell ALL patients only.

**Table 3:** Treatment outcomes

Outcome variable	5-years OS ± SE	*p*	5-years EFS ± SE	*p*	5-years CIR ± SE	*p*
Overall	89.5 ± 4.0%		87.6 ± 4.3%		7.1 ± 3.5%	
B-cell ALL	90.5 ± 2.4%		88.7 ± 2.6%		6.4 ± 2.0%	
T-cell ALL	86.0 ± 6.2%		83.2 ± 7.2%		7.4 ± 4.0%	
NCI risk		≤ 0.05		≤ 0.05		
Standard risk	94.2 ± 2.0%		91.9 ± 2.4%		5.4 ± 1.9%	
High risk	81.6 ± 6.1%		81.2 ± 6.0%		8.9 ± 3.8%	
WBC count		≤ 0.05		≤ 0.05		≤ 0.05
< 50 × 10^9^/L	94.5 ± 1.9%		92.3 ± 2.3%		5.2 ± 1.8%	
≥ 50 × 10^9^/L	79.0 ± 7.1%		78.5 ± 7.0%		10.3 ± 4.5%	
Extra-medullary status, B-cell						≤ 0.05
No	90.8 ± 2.4%		88.8 ± 2.7%		6.5 ± 2.1%	
Yes	88.4 ± 10.8%		86.0 ± 8.9%		4.9 ± 5.2%	
Extra-medullary status, T-cell						
No	89.3 ± 6.6%		86.0 ± 7.6%		3.6 ± 3.7%	
Yes	73.3 ± 18.1%		72.7 ± 18.5%		27.3 ± 18.5%	
Cytogenetic subtype		≤ 0.05				
Normal	92.8 ± 4.4%		90.5 ± 4.7%		4.6 ± 2.9%	
*ETV6/RUNX1*	95.9 ± 4.2%		92.7 ± 5.4%		3.5 ± 3.6%	
Hyperdiploidy	93.1 ± 3.3%		90.1 ± 3.7%		5.6 ± 2.6%	
*MLLR*	60.7 ± 34.6%		55.4 ± 29.6%		25.8 ± 22.1%	
*t*(1;19)/*TCF3*-*PBX1*	65.0 ± 31.5%		58.7 ± 25.8%		31.2 ± 23.7%	
Others	90.3 ± 4.3%		88.5 ± 4.4%		6.5 ± 3.1%	
Cytogenetic group		≤ 0.05		≤ 0.05		≤ 0.05
*MLLR* + t(1;19)	63.1 ± 23.6%		57.2 ± 20.2%		29.5 ± 17.1%	
All others	92.5 ± 2.1%		90.0 ± 2.5%		4.4 ± 1.8%	
Day 15 bone marrow		≤ 0.05		≤ 0.05		≤ 0.05
M1	92.5 ± 2.1%		91.1 ± 2.3%		5.1 ± 1.7%	
M2	63.0 ± 26.9%		55.9 ± 29.6%		34.6 ± 25.5%	
M3	46.9 ± 53.9%		40.1 ± 54.1%		23.8 ± 27.8%	
Day 15 MRD		≤ 0.05		≤ 0.05		≤ 0.05
< 0.01%	93.6 ± 2.8%		91.9 ± 3.1%		5.6 ± 2.5%	
≥ 0.01%	79.1 ± 7.4%		74.7 ± 8.3%		15.0 ± 6.2%	
MRD transition (D15 → D29)		≤ 0.05		≤ 0.05		
N → N	94.6 ± 2.6%		92.9 ± 2.9%		5.7 ± 2.5%	
Y → N	83.1 ± 7.2%		77.4 ± 8.4%		16.0 ± 7.0%	
Y → Y	71.2 ± 20.1%		71.1 ± 20.1%		10.0 ± 11.0%	
Rapidity of response		≤ 0.05		≤ 0.05		≤ 0.05
RER	92.8 ± 2.1%		91.5 ± 2.3%		5.2 ± 1.8%	
SER	72.3 ± 14.7%		66.0 ± 16.2%		20.0 ± 11.8%	
Post-induction regimen		≤ 0.05				
Arm A	94.0 ± 2.3%		91.8 ± 2.6%		5.7 ± 2.1%	
Arm B	91.1 ± 4.8%		89.5 ± 4.8%		2.9 ± 2.2%	
Arm C	80.1 ± 7.6%		79.3 ± 7.5%		12.0 ± 5.4%	
Arm assignment history		≤ 0.05		≤ 0.05		≤ 0.05
A → A	94.2 ± 2.2%		92.5 ± 2.5%		5.1 ± 2.0%	
A → C	90.3 ± 10.2%		83.1 ± 13.2%		8.0 ± 8.5%	
B → B	93.5 ± 4.0%		91.9 ± 4.0%		2.6 ± 2.0%	
B → C	56.7 ± 0.26%		52.2 ± 27.1%		41.4 ± 25.1%	
C → C	93.6 ± 6.3%		91.6 ± 4.8%		2.5 ± 2.6%	
Gender						
Female	86.7 ± 4.1%		85.4 ± 4.2%		6.6 ± 2.6%	
Male	92.5 ± 2.8%		90.4 ± 3.1%		7.7 ± 2.8%	
Gender vs. treatment arm, female		≤ 0.05		≤ 0.05		≤ 0.05
Arm A	93.6 ± 3.4%		91.1 ± 3.7%		5.9 ± 3.0%	
Arm B	86.0 ± 8.2%		85.3 ± 7.2%		2.6 ± 2.7%	
Arm C	71.3 ± 15.3%		69.7 ± 15.1%		17.2 ± 11.3%	
Gender vs. treatment arm, male						
Arm A	94.4 ± 3.0%		92.4 ± 3.2%		6.8 ± 3.1%	
Arm B	100.0 ± 0.0%		95.4 ± 4.8%		4.4 ± 4.6%	
Arm C	85.7 ± 7.9%		85.1 ± 7.5%		11.5 ± 6.6%	
Down syndrome						
No	90.8 ± 2.4%		88.8 ± 2.7%		6.8 ± 2.1%	
Yes	89.0 ± 10.8%		85.9 ± 8.3%		0.0%	

*OS* overall survival, *EFS* event-free survival, *CIR* cumulative incidence of relapse, *SE* standard error, *p*, p-value, only significant values are shown. Extra-medullary status: *No* no extra-medullary disease. Yes, extra-medullary (CNS, testes) site involved. *MLLR*, MLL rearrangement, *RER* rapid early response, *SER* slow early response, *MRD* minimal residual disease. MRD transition (D15 → D29), MRD kinetics from day 15 to day 29 of induction: “N → N”: day 15 MRD % < 0.01%, day 29 < 0.01%; “Y → N”: day 15 ≥ 0.01%, day 29 < 0.01%; “Y → Y”: day 15

### Treatment outcomes

**Fig. 3:**
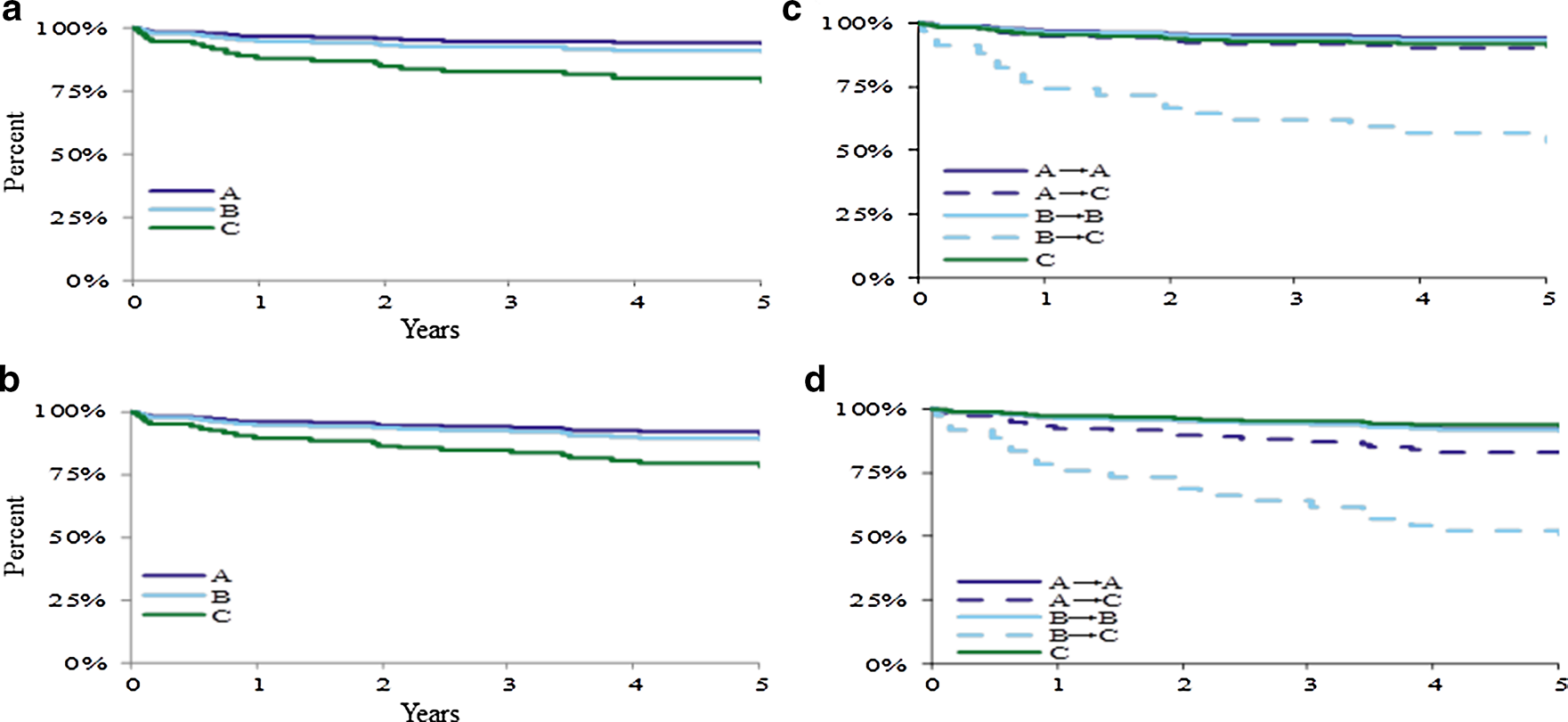
Overall survival (OS) and event-free survival (EFS) by treatment regimen. The 5-year OS based on post-induction treatment regimen (**a**) and arm reassignment history (**c**), and the 5-year EFS based on post-induction treatment regimen (**b**) and arm reassignment history (**d**). Patients treated with Arm C had significantly inferior (p < 0.05) OS (**a**). Patients who were NCI high-risk and treated with induction Arm B but reassigned due to slow early response (SER) to post-induction Arm C (B → C, depicted in the light dashed line in **c**, **d**) had significantly (p < 0.05) inferior survival outcomes despite treatment intensification reassignment. In contrast, NCI standard-risk patients treated with induction Arm A and then reassigned for SER to post-induction Arm C (A → C) had favorable outcomes comparable to those of patients with rapid early response treated with regimen Arm A (**c**, **d**)

Post-induction treatment arm was significantly associated with survival outcomes (Table 3, Fig. 3). Significant pairwise differences in OS and EFS by treatment were found between Arm A vs. C (p ≤ 0.05).

### Potential prognostic factors

Clinical features (NCI-risk, WBC level, extramedullary involvement by phenotype, gender, and co-existence of DS), cytogenetic subtype/group, and response criteria were modeled vs. outcome, independent of treatment regimen, as potential prognostic factors (Table 3). The impact of treatment intensification based on history of arm (re)assignment was also modeled vs. outcome to study the effect of treatment intensification on outcome (Table 3).

### Clinical features vs. outcome

NCI-risk had significant effects on OS and EFS for B-cell patients with inferior survival outcomes for NCI-HR vs. NCI-SR patients (Table 3). However, when NCI-risk and treatment arm were modeled together, NCI-HR patients treated with Arm C had worse outcomes than those receiving Arm B. Given that treatment Arm B segregated completely into NCI-HR, and those patients with poor response on Arm B were then transferred to Arm C, this may reflect limited positive response of NCI-HR to the form of intensification used in Arm C (Fig. 3).

Similarly, WBC ≥ 50,000 at diagnosis was significantly associated with poor prognosis for OS, EFS, and CIR (Table 3). However, when treatment and WBC level were modeled together, a significant interaction was found for all outcome measures and treatment for high WBC (≥ 50 × 10^9^) patients. Specifically, treatment Arm C significantly associated with negative outcomes for those patients with WBC ≥ 50, which corresponded to the negative effect of SER.

Extra-medullary disease involvement at diagnosis may constitute a clinically unique subset of ALL patients. To test this hypothesis, we tested outcomes in B- and T-cell phenotype vs. the combined set of patients with CNS3 and/or patients with testicular involvement. No B-cell patients had a significant association between extra-medullary status and any outcome (Table 3). However, in T-cell patients (Table 3), we found a significant association between extra-medullary status and greater rates of relapse (HR = 8.62, p ≤ 0.05). This was the only significant finding for T-cell patients in our study.

**Fig. 4:**
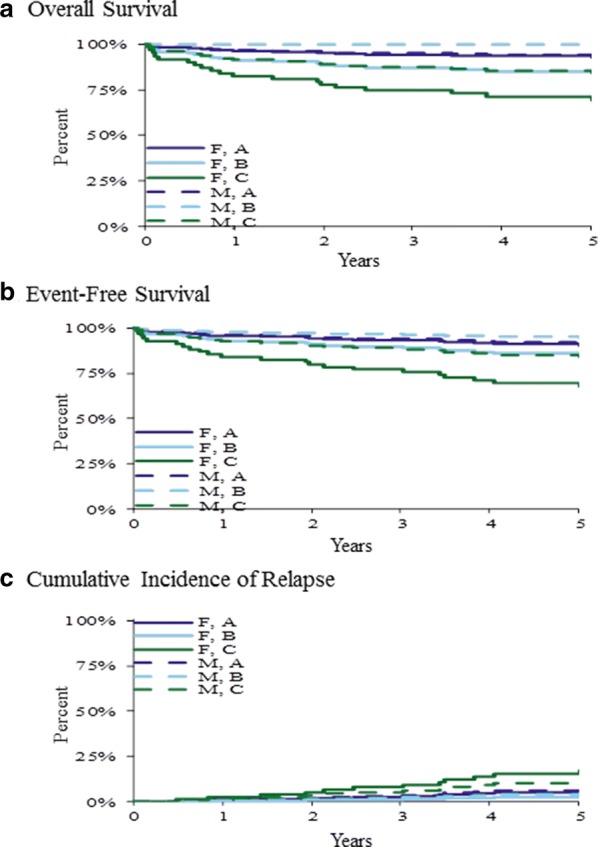
Treatment outcomes by gender and treatment regimen. The 5-year overall survival (**a**), event-free survival (**b**), and cumulative incidence of relapse (**c**) by gender and treatment arm. Significant differences (p < 0.05) were observed in overall survival with inferior outcome observed in female patients treated with Arm B (F, B) and Arm C (F, C) vs. male patients treated with the same (M, B and M, C) regimens. In contrast, female vs. male patients treated with the less intensive regimen Arm A (F, A vs. M, A) had similar overall survival outcome

Overall, no difference in outcomes was observed by gender (Table 3). However, when gender and treatment were modeled together (Table 3, Fig. 4), a significant (p ≤ 0.05) overall effect was seen for OS. Specifically, OS was significantly inferior in female patients treated with treatment Arms B and C compared to male patients treated with the same arms. In contrast, no gender-related difference was observed in patients treated with the less intense Arm A regimen.

None of the patients with DS had T-cell ALL. Furthermore, no differences in outcomes were observed in B-cell ALL patients with or without DS (Table 3).

### Cytogenetic effects on outcomes

**Fig. 5:**
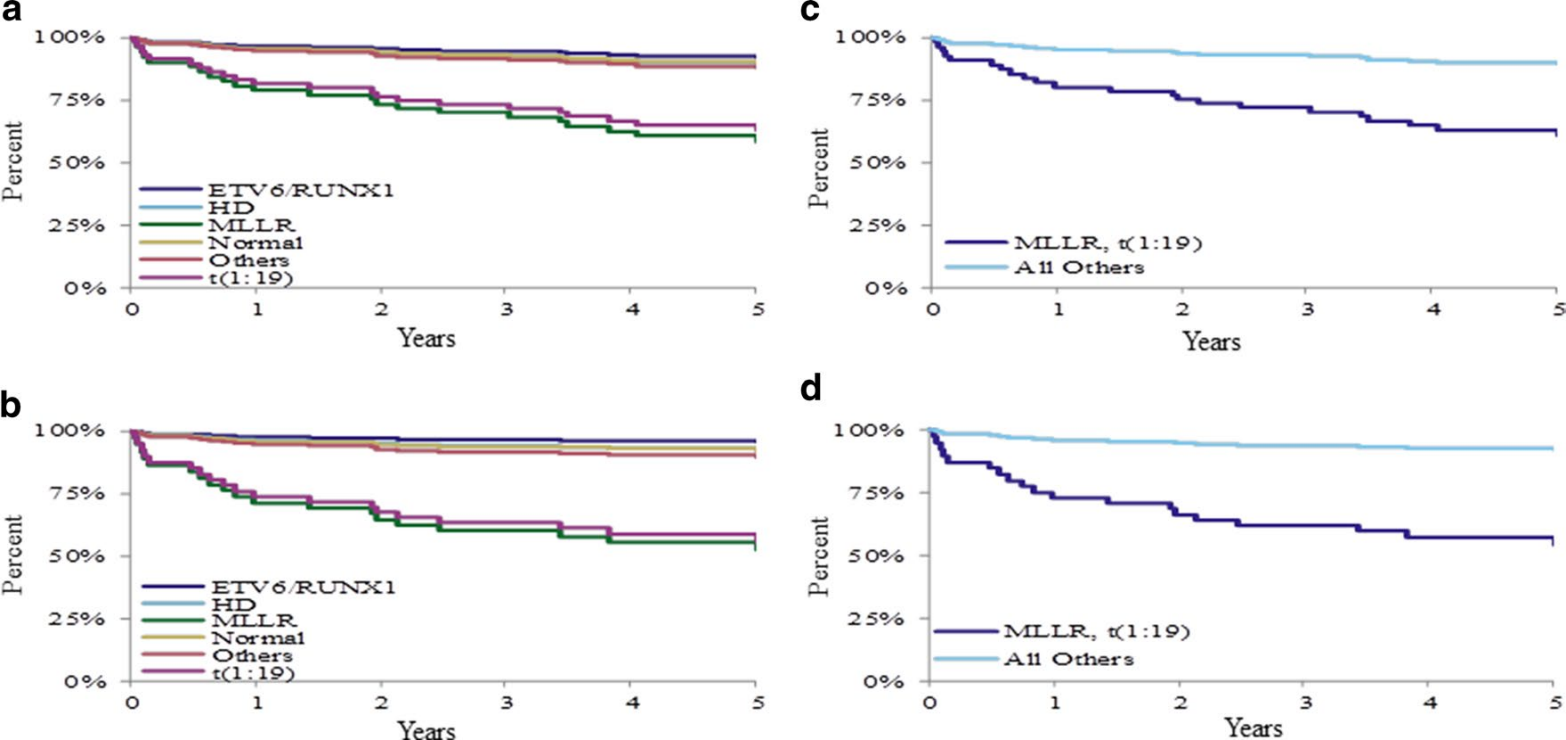
Outcomes by cytogenetic type and subgroup, B-cell phenotype. The 5-year overall survival by genetic subtype (**a**) and subgroup (**c**), and the 5-year event-free survival by genetic subtype (**b**) and subgroup (**d**) are shown. The overall survival and event-free survival of the *t*(1;19) cytogenetic abnormality and the *MLL*-rearrangement (MLLR) clustered distinctly away from all other genetic subtypes (**a**, **b**). Patients with the *t*(1;19) and MLLR subgroup had significantly (p < 0.05) inferior survival outcomes compared to all other genetic subtypes (**c**, **d**)

Cytogenetics had a significant effect on OS for B-cell patients (Table 3). Both *MLLR* and *t*(1:19) clustered distinctly away from all other cytogenetic results (Fig. 5a, b) in regard to survival. Thus grouping of cytogenetic types (*MLLR* + *t*(1:19) vs. all others) was modeled vs. outcome, significant results were found for all outcomes in B-cell patients (Table 3, Fig. 5c, d).

Patients with favorable cytogenetic features ((hyperdiploidy and ETV6/RUNX1) were found more frequently in the B → B and C → C treatment groups than in the B → C treatment group (27/94 vs. 0/12, Fisher’s exact test, two-tailed p value = 0.034).

### Rapidity of response as a prognostic factor

**Fig. 6:**
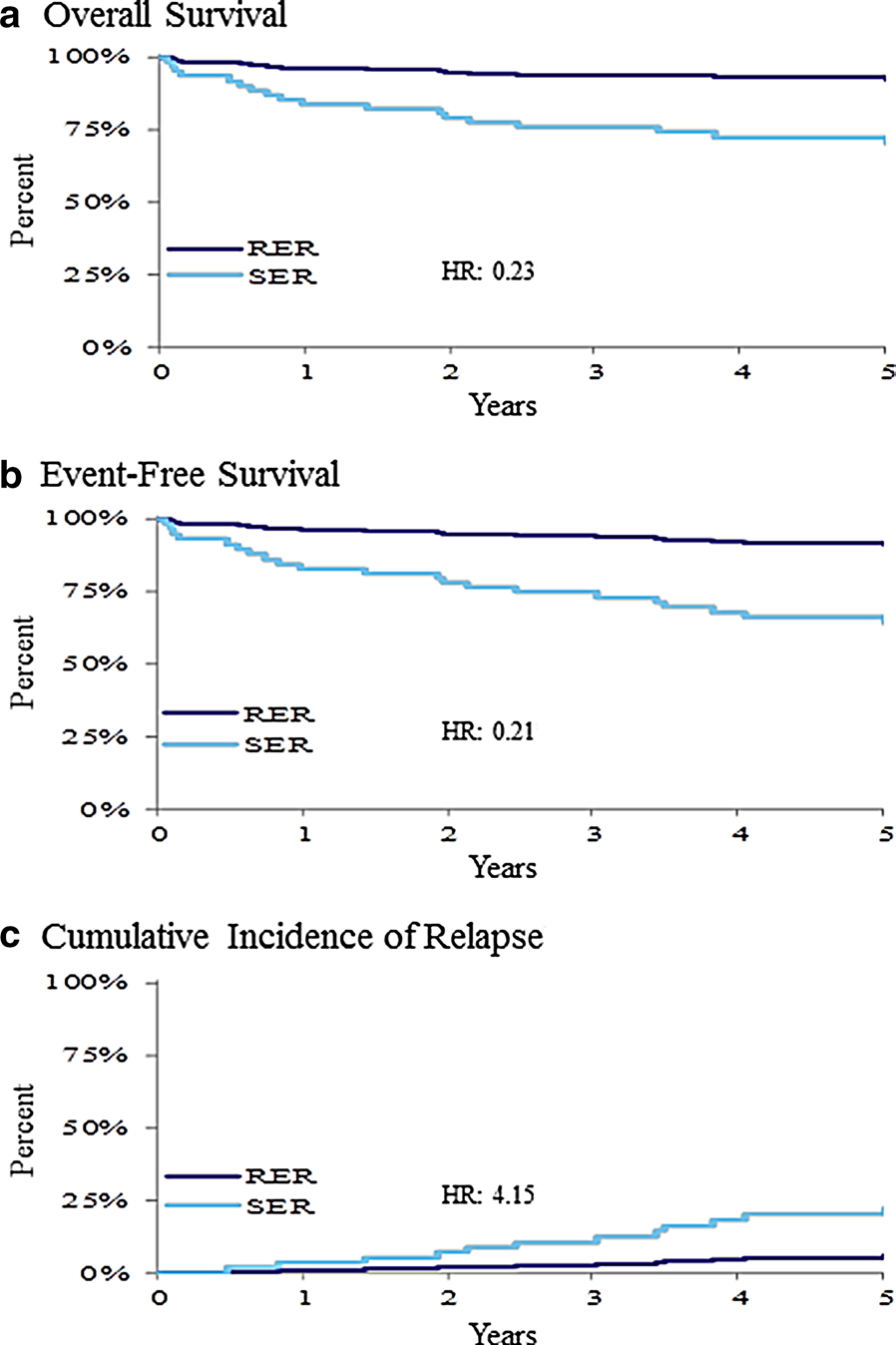
The 5-year overall survival (**a**), event-free survival (**b**), and cumulative incidence of relapse (**c**) based on response: rapid early response (RER) vs. slow early response (SER). Patients with SER had significantly (p < 0.05) worse results for all three outcome measures. *HR* hazard ratio

Bone marrow by morphology at day-15 (M1, vs. M2, vs. M3, Table 3) was prognostic. Day-29 marrow could not be analyzed as too few samples remained at M2 or M3. Response (RER vs. SER) had a distinct and significant effect on all outcomes for B-cell patients (Table 3, Fig. 6). SER was associated with inferior outcomes.

### Evaluation of MRD and outcome

**Fig. 7:**
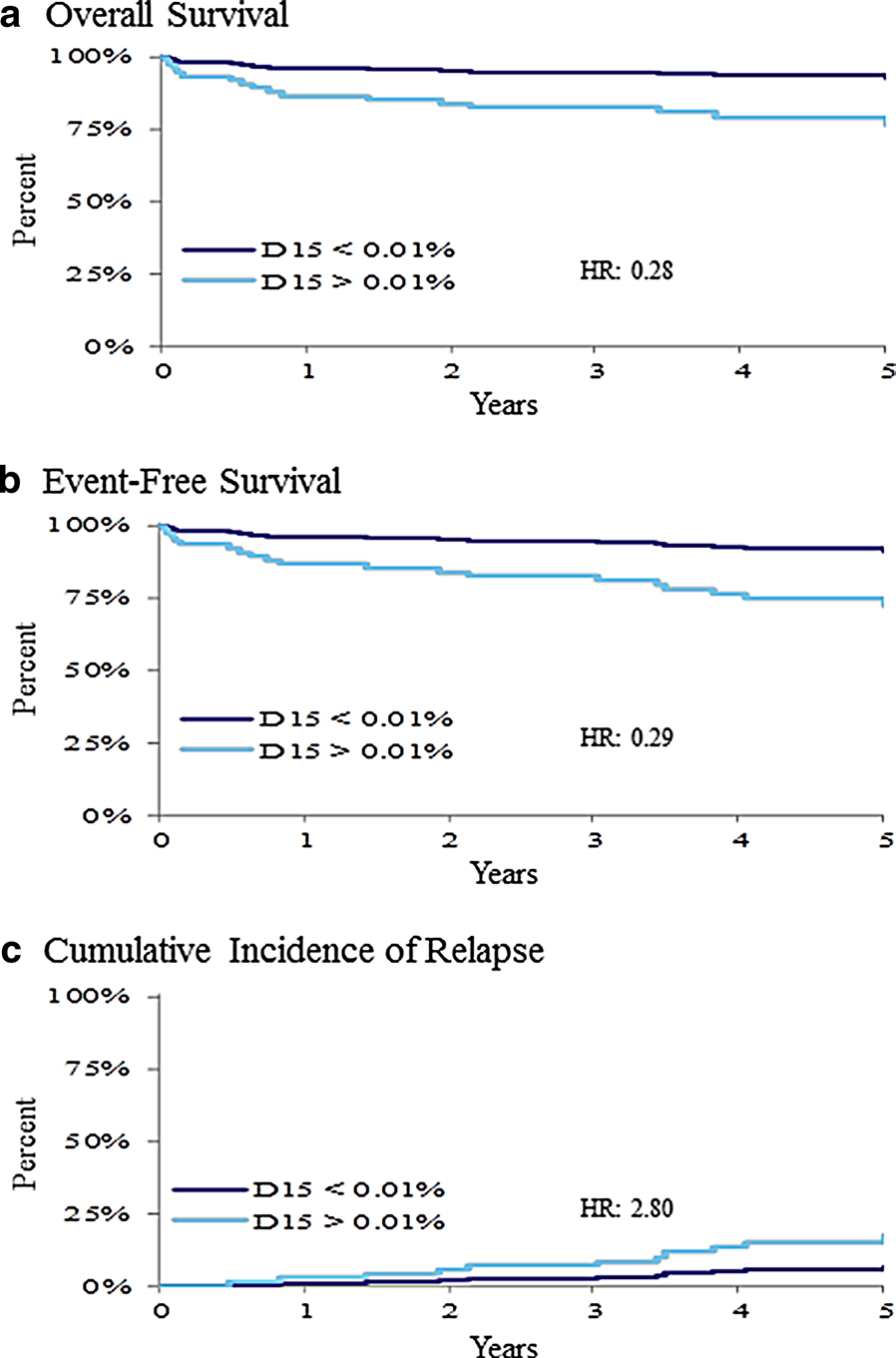
The 5-year overall survival (**a**), event-free survival (**b**), and cumulative incidence of relapse (**c**) based on day 15 minimal residual disease (MRD) response: MRD negative (< 0.01%) vs. MRD positive (≥ 0.01%). Patients with day-15 MRD positive had significantly (p < 0.05) worse results for all three outcome measures. *HR* hazard ratio

**Fig. 8:**
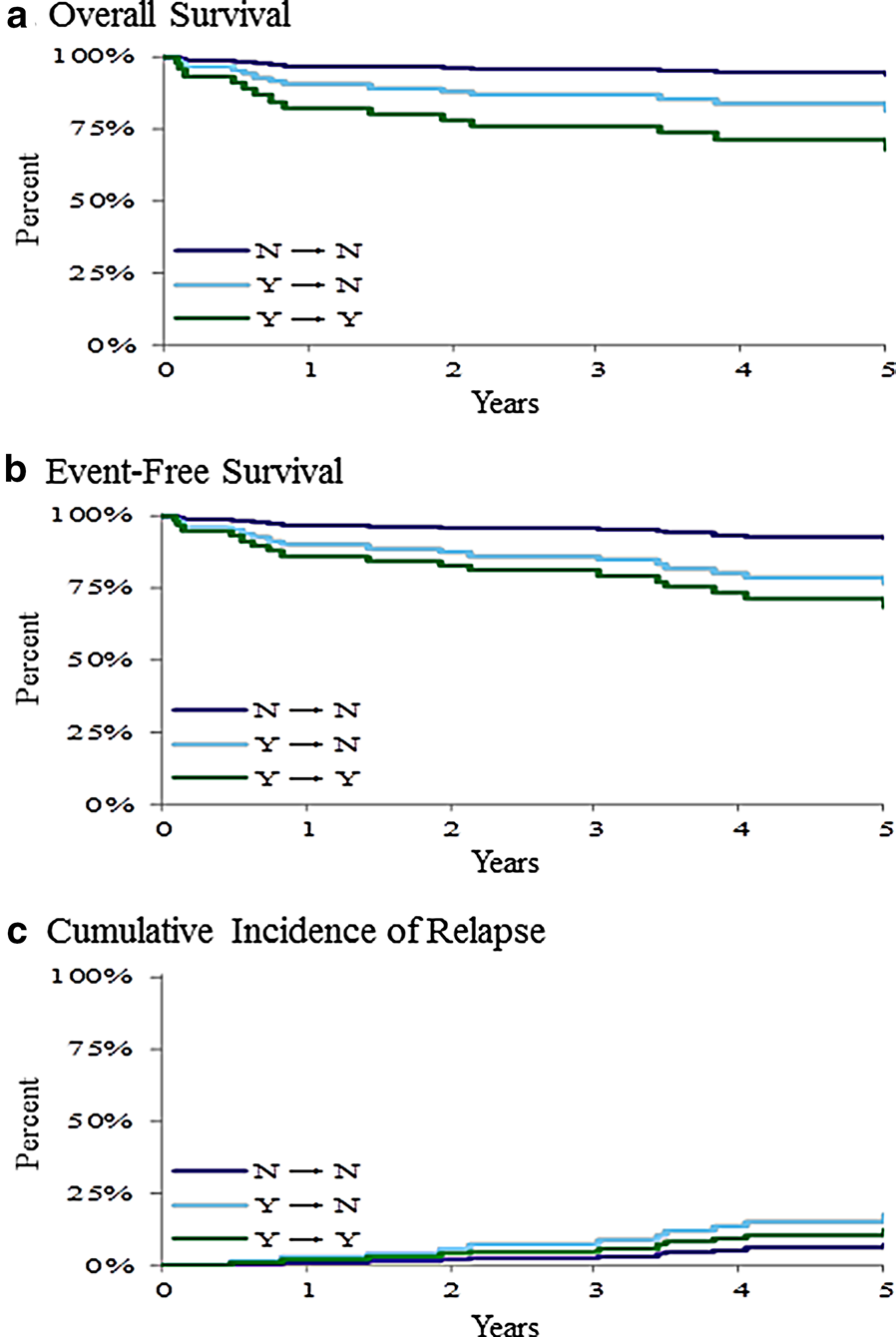
The 5-year overall survival (**a**), event-free survival (**b**), and cumulative incidence of relapse (**c**) based on minimal residual disease (MRD) transition from day-15 to day-29 of induction. N → N, day 15 MRD negative (< 0.01%) and day-29 MRD negative (< 0.01%). Y → N, day-15 MRD positive (≥ 0.01%) but day-29 MRD negative (< 0.01%). Y → Y, day-15 MRD positive (≥ 0.01%) and day-29 MRD positive (≥ 0.01%). Survival outcome differences were statistically significant (p < 0.05)

**Fig. 9:**
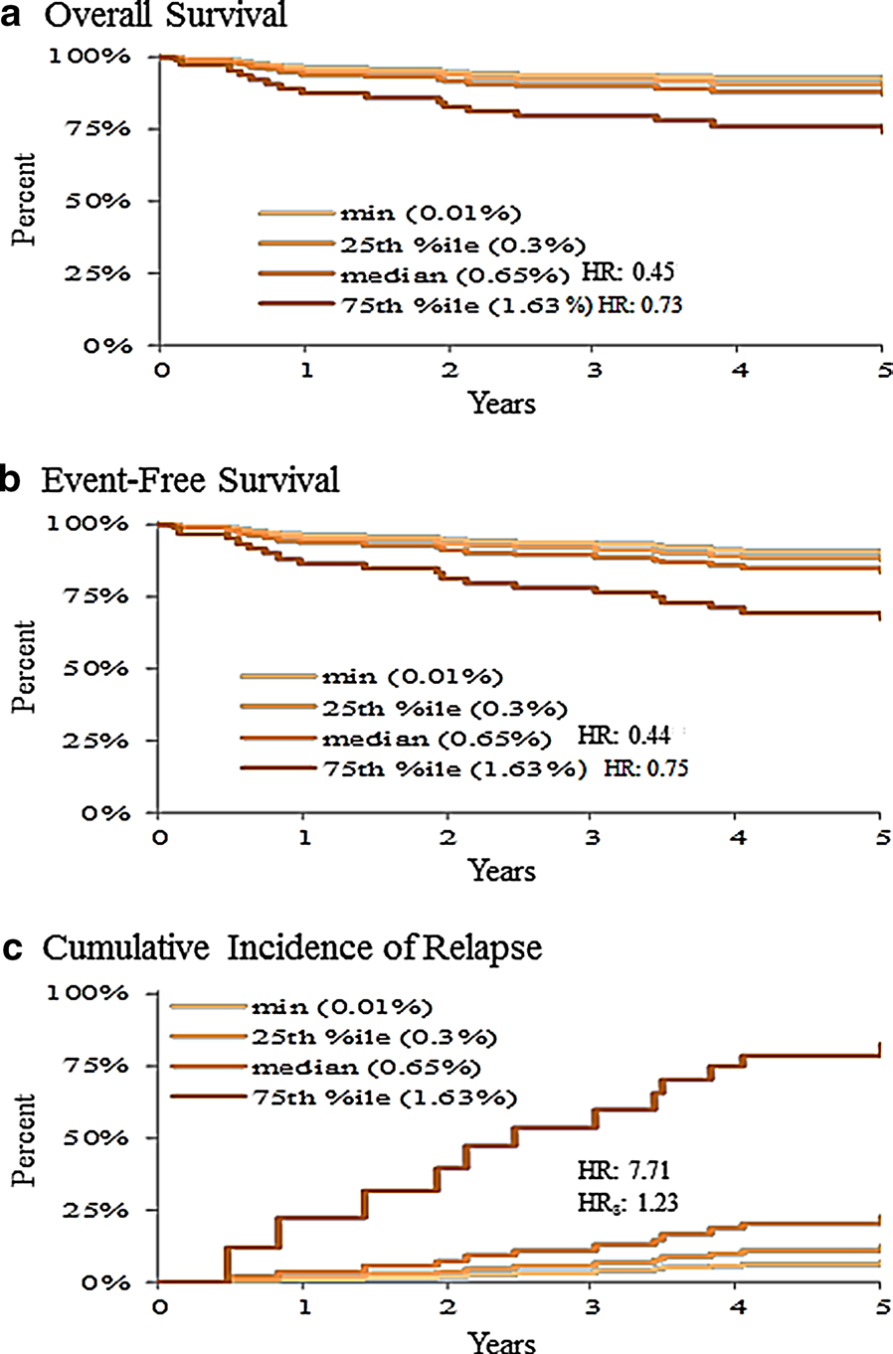
The 5-year overall survival (**a**), event-free survival (**b**), and cumulative incidence of relapse (**c**) based on day-29 (end-of-induction) minimal residual disease (MRD) level (percent scores) > 0.01%. *HR* hazard ratio

Negative MRD at day-15 (Table 3) associated with better prognosis in B-cell patients for all outcomes (Fig. 7). When MRD transitions were modeled (Table 3), significant differences were found for OS and EFS in B-cell patients (Fig. 8). When MRD percent scores were modeled vs. outcomes, treating negative/positive MRD status as a nuisance variable, no significant association was found between MRD% (above 0.01) at day-15 and outcomes, but significant associations were found between MRD% (above 0.01) at day-29 and poor prognosis for OS and EFS (Fig. 9). While the only significant pairwise comparisons were between day-15 negative vs. either of the day-15 positive starting states, those patients who did not achieve a negative MRD by end-of-induction tended to have worse outcomes (Table 3).

### Treatment (re)assignment history vs. outcome

**Fig. 10:**
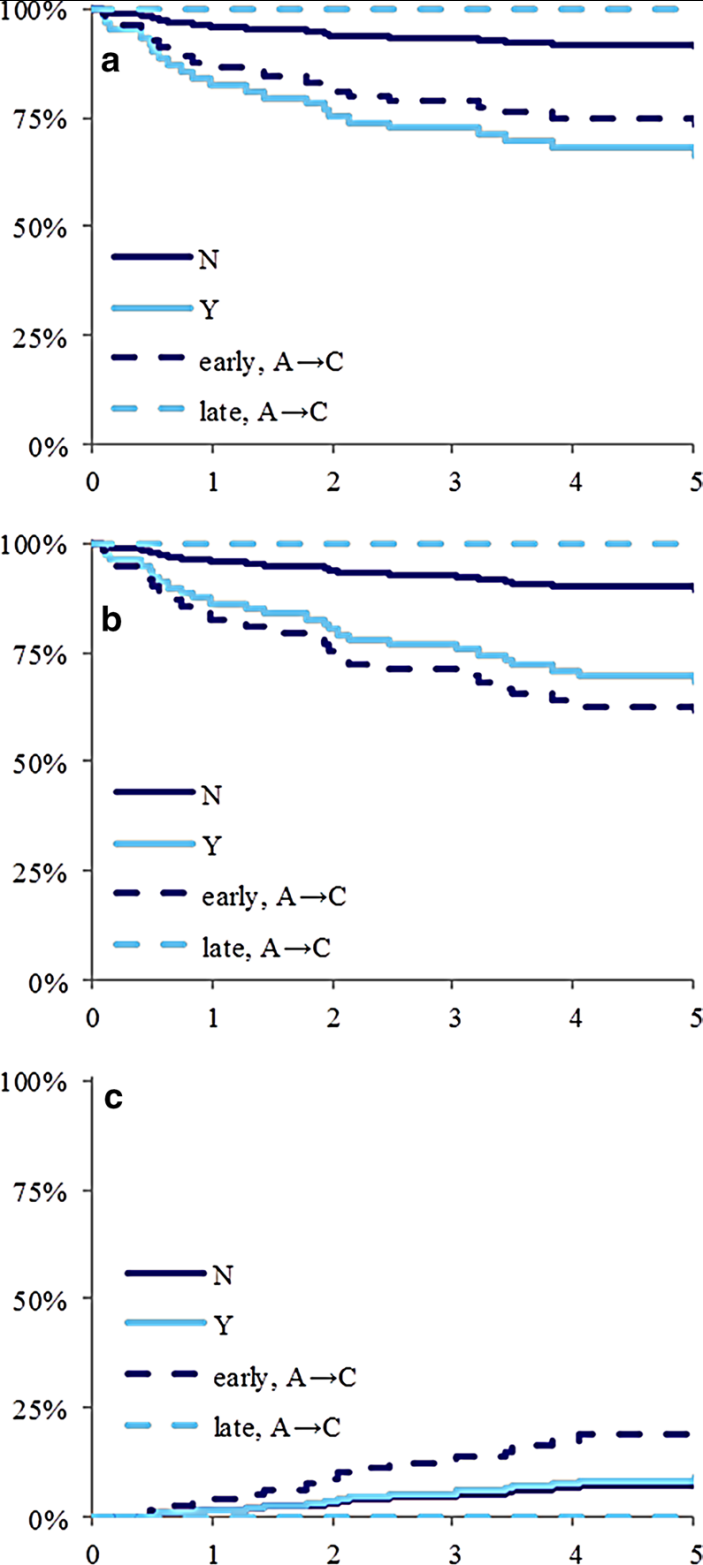
The 5-year overall survival (**a**), event-free survival (**b**), and cumulative incidence of relapse (**c**) by “late” vs. “early” Arm A → C patient reassignments, B-cell phenotype, treating “early, A → C” and “late, A → C” reassigned patients as separate stratifications. “N”: Day 29 MRD < 0.01%, no reassignment (solid dark blue line); “Y”: Day 29 MRD ≥ 0.01% and no reassignment (solid light blue line). All “N” subjects were Arm A induction (i.e. A → A group). All “Y” subjects were Arm C induction (i.e. C → C group but MRD ≥ 0.01% at day 29). “early, A → C”, which is depicted as a dashed dark blue line represents reassigned NCI-SR patients to Arm C early due to ≥ 5% bone marrow blasts at day 15 of induction; “late, A → C”, which is depicted as a dashed light blue line represents reassigned NCI-SR patients to post-induction Arm C due to < 5% blasts at day 15 but MRD ≥ 0.01% at day 29 of induction. Both overall survival and event-free survival showed a significant (p ≤ 0.05) effect if patients who began treatment on Arm A but were switched to Arm C were stratified separately as “early” vs. “late”. The “late, A → C” group had significantly better outcomes, which were similar to patients assigned to Arm A and had rapid early response at both assessment time points on day 15 and 29 of induction (A → A group)

To understand the complex relationship of MRD and outcome, we compared outcomes of patients grouped by treatment assignment history based on induction treatment assignment and post-induction response-based reassignment (Table 3). Patients assigned to induction Arm A were NCI-SR while patients assigned to induction Arm B were NCI-HR. Treatment reassignment for these patients to the more intensive Arm C was based on response. Thus, patients with NCI-SR and SER were reassigned to Arm C (A → C) and patients with NCI-HR and SER were reassigned to Arm C (B → C). Of note, the Arm B → C subgroup had worse treatment outcomes than all other groups (Table 3, Fig. 3c, d). In contrast, the Arm A → C subgroup had similar outcomes to the Arm A → A subgroup (Table 3, Fig. 3c, d). However, this analysis included patients who had begun on induction Arm A and switched after day 15 (for slow response) to continue on induction and post-induction treatment Arm C. We, therefore, modeled the subset of patients for whom treatment arm was switched from A to C as a separate level. Two such patient groups existed, early and late. The early group was switched on day 15 if they had marrow blast levels M2 or M3 (≥ 5%) regardless of day 29 MRD. The late group was switched on day 29 if they were MRD positive. These two groups were then stratified based on the timing of treatment intensification (early A → C vs. late A → C, see Fig. 10). Patients treated with late A → C had similar outcomes to patients assigned to Arm A and had a rapid early response at both assessment time points on day 15 and 29 of induction (A → A group). Notably, the late switched group had significantly better outcomes with 100% 5-year OS and EFS and 0% CIR while early switched patients had a worse prognosis for all measures.

### Death as first event (DAFE)

DAFE was analyzed as a crude estimate for treatment toxicity. DAFE (induction death and death in CR) occurred in 13 patients. The 5-year cumulative incidence of DAFE was 6.3 ± 1.69%. There was no difference in the cumulative incidence of DAFE by age at diagnosis. However, female patients had a significantly worse 5-year cumulative incidence of DAFE compared to male patients (11.2 ± 3.34% vs. 2.5 ± 1.45%; p = 0.021).

A total of 4 patients died during induction. The 5-year cumulative incidence of induction death was 1.03 ± 0.84%. Induction death was higher in older (≥ 10 years) than younger (< 10 years) patients (7.1 ± 4.87% vs. 1.0 ± 6.73%, p = 0.016). No difference in induction death was observed by gender or induction regimen used.

Death in CR occurred in 9 patients. The 5-year cumulative incidence of death in CR was 4.0 ± 1.32%. The 5-year cumulative incidence of death in CR was higher for patients treated with post-induction regimen B than for those treated with post-induction regimen A (7.3 ± 4.07 vs. 0.9 ± 8.97%; p = 0.026). The 5-year cumulative incidence of death in CR was higher for patients treated with post-induction regimen C than for those treated with post-induction regimen A (7.1 ± 3.08 vs. 0.9 ± 8.97%; p = 0.023). There was no difference in the 5-year cumulative incidence of death in CR of patients treated with post-induction regimens B and C. The 5-year cumulative incidence of death in CR was greater for female than male patients (7.4 ± 2.70% vs. 1.5 ± 1.08%; p = 0.03). There was no difference in the 5-year cumulative incidence of death in CR by patient age at diagnosis.

## Discussion

Two hundred and forty-one children with ALL were assigned to one of three treatment arms of increasing intensity depending upon specific risk factors and responses described herein. Patients in the first two arms were evaluated for possible reassignment to the most intensive regimen on days 15 and 29 of induction. Our outcomes compare well with those of leading leukemia cooperative groups. For COG AALL0331 (SR only), 5-year continuous OS was 98.8%, EFS was 96.4% and CIR was 4.8% [19]. In the present study, NCI-SR patients had a 5-year OS of 94.2% ± 2.0%, EFS of 91.9% ± 2.4%, and CIR of 5.4% ± 1.9%. NCI-HR patients in COG AALL0232 had 5-year OS of 85.0% ± 0.9% and EFS of 75.3% ± 1.1%, compared to our patients OS of 81.6% ± 6.1% and EFS of 81.2% ± 6.0% [8].

Patients with T-cell ALL treated with COG AALL0434 with HDMTX had a 4-year disease-free survival of 86.1% ± 2.4% [13]. Patients we treated with our similar T-cell ALL regimen had a 5-year EFS of 85.7% ± 6.2%. The only prognostic factor identified for patients with T-cell ALL in our study was the presence of extra-medullary disease.

Extra-medullary disease outcomes suggest that intensification of chemotherapy for extra-medullary disease improves outcomes in B-cell ALL but not in T-cell ALL. Outcome by cytogenetic group identified a poor risk cluster that included *MLLR* and *t*(1;19). The *t*(1;19) abnormality was at one time associated with poor prognosis, but refinements in treatment have improved the prognosis [20]. This cytogenetic group is also associated with other high-risk factors, such as high WBC and absence of hyperdiploidy [20]. Our cohort size was not large enough to explore whether our patients with *t*(1:19) ALL significantly overrepresented these other risk factors. Population-specific effects of known risk factors exist for diverse disorders, including ALL. Disparity in treatment outcome could reflect ethnicity-related genetic variation, since ethnic and racial disparities in response to ALL treatment regimens are well known [21]. Disparity in outcomes by genetic subtype in childhood acute myeloid leukemia exist in our population [22]. Therefore, further genetic studies in children with ALL in our population are warranted.

We intensified post-induction therapy based on SER, using the same post-induction regimen for NCI-SR patients who began on Arm A induction (A → C) and for NCI-HR patients who began on Arm B induction (B → C). Only intensification in patients with NCI-SR ALL was associated with positive outcomes. Despite comparable high-risk clinical characteristics in patients eligible for induction Arm B, SER patients reassigned to Arm B → C had inferior OS, EFS, and CIR compared to RER patients who remained on Arm B → B. Similarly, despite additional high-risk clinical features including extramedullary disease and steroid pretreatment in patients assigned to the C → C group, patients in the B → C group had inferior outcomes. Genetics of NCI risk levels show that, particularly within HR subjects, specific genotypes play a significant independent role in patient outcomes [23]. Thus, SER in the present study could reflect the biology of leukemia. In fact, patients in the B → C group had less frequent occurrence of favorable cytogenetic features compared to the B → B and C → C group. This underscores the fact that assigning/reassigning patients using a single intensification approach is suboptimal, specifically given that different genotypes of ALL respond differently to this assignment method [9].

MRD had complex associations with outcome in our study. Day-15 MRD status (negative vs. positive) was prognostic in all treatment groups and end-of-induction MRD level at day 29 was also prognostic. This finding agrees with a large study that reported day-29 MRD as the strongest prognostic factor [4]. Day-15 to day-29 MRD transition was also prognostic. We intensified therapy for those patients who began on Arm A but either had ≥ 5% blasts on day 15 (early intensification) or < 5% blasts on day 15 but did not convert to MRD% < 0.01 by day 29 (late intensification). Early intensification was not associated with improved outcomes. In contrast, late intensification on the basis of end-of-induction MRD ≥ 0.01% was associated with a 100% OS and EFS, and 0% CIR. Despite the limited number of patients in each subgroup, our study suggests that a single time-point for intensification is not informative and kinetics of MRD over different time points need to be considered in order to optimize patient outcomes.

The threshold level of MRD that would benefit from treatment intensification at early time points during induction needs to be evaluated further. The present study showed that patients with ALL who had MRD-positivity at the end-of-induction, but achieved MRD-negativity at the end of consolidation, benefited from continuing post-induction standard chemotherapy, particularly if they were NCI-SR (A → C). However, patients with NCI-HR and MRD-positivity at the end of induction (B → C), or those with high level (≥ 5%) MRD at earlier time points (day 15 induction), regardless of NCI-risk (early A → C), faired poorly when treated with post-induction standard chemotherapy, compared to other treatment groups. Furthermore, our study showed that higher levels (> 1%) of MRD at the end-of-induction were associated with a significantly increased cumulative incidence of relapse (Fig. 9). Thus, optimization of post-induction therapeutic approaches is needed. Early identification of patients at high risk for relapse based on MRD may optimize timely introduction of emerging therapies that have recently shown to improve outcomes and possibly cure rates particularly for patients with MRD-positivity at the end of induction [24, 25]. The results of this study suggest time points to introduce these newer therapies.

We found no difference in treatment toxicity by treatment regimen when using DAFE as a crude estimate of treatment toxicity. However, differences in gender-related toxicity were observed in our patients, apparently due to a higher incidence of DAFE in females. This finding was mainly related to a higher cumulative incidence of remission deaths in females receiving high intensity regimens (Arm B and C regimens). The cause for gender related differences in treatment related toxicity is not clear, but may be due to gender-related pharmacokinetic differences suggested in our population [26]. Our observations are supported by those reported by the Children’s Oncology Group study that showed a higher likelihood of treatment-related death in females undergoing treatment for high-risk ALL [27]. No significant difference in treatment outcomes were observed in patients with Down syndrome, compared to those without Down syndrome in our study.

Our study was a single-center prospective study administered by a single governing entity. In assessing effects of diseases such as ALL, single-center studies may be inadequate to determined and explain potentially unique influences on treatment outcomes and to find optimized treatments tailored to potentially unique genetics and environmental influences in a region. Despite these limitations, our study has helped close a gap in knowledge of treatment outcomes for leukemia in affluent developing countries. This work has shown that any gap between the “developed” and “developing” world in cancer treatment can be closed by appropriate application of resources. The present study confirmed that conducting a prospective clinical study in our setting is feasible. In fact, implementing a clinical trial-based approach that utilizes a risk and response-based protocol produced comparable outcomes to those reported by leading leukemia cooperative groups.

## Conclusions

In this prospective study, we evaluated effects of risk-based treatment intensification by minimal residual disease assessment at different time points, including intensification of therapy based on response assessment at day-15 and MRD at day-29 of induction to test if treatment intensification would improve outcomes. Results showed that MRD level at end-of-induction associated with outcomes, but association with a specific MRD value at end-of-induction varied significantly by NCI-risk group. Although treatment intensification improved outcomes of NCI-SR patients with positive MRD at end-of-induction, further refinement is needed to improve outcomes of patients presenting with NCI-HR and slow early response. Assigning patients by end-of-induction MRD-risk alone did not reflect response kinetics of the different NCI-risk groups. Integration of NCI-risk group with specific MRD value and time point allows more refined treatment stratification.

## Abbreviations

ALL: acute lymphoblastic leukemia
CALL08: childhood acute lymphoblastic leukemia 2008
CIR: cumulative incidence of relapse
CNS: central nervous system
DAFE: death as first event
DS: down syndrome
EFS: event-free survival
FISH: fluorescence in situ hybridization
HD: hyperdiploidy
HDMTX: high-dose methotrexate
HR: high risk
iAMP21: intrachromosomal amplification of chromosome 21
IT: intrathecal
KAIMRC: King Abdullah International Medical Research Center and Ethics Review Committee
KAMC: King Abdulaziz Medical City
LAIP: leukemia-associated immunophenotypes
LR: low risk
LSD: least-significant difference
*MLLR*: MLL cytogenic rearrangement
MRD: minimal residual disease
NCI: National Cancer Institute—Rome
OS: overall survival
PNOC: Princess Noorah Oncology Center
RER: rapid early response
SER: slow early response
SR: standard risk
